# Modeling of thermal errors for dual-spindle turning-milling compound machine tools based on hybrid networks

**DOI:** 10.1038/s41598-025-27599-1

**Published:** 2025-12-30

**Authors:** Wentao Lu, Jianchen Wang, Jianqiang Zhou, Xiaolei Deng, Hongyi Wu, Lei Zhang

**Affiliations:** 1 Zhejiang Provincial Key Laboratory of Intelligent Manufacturing for Aerodynamic Equipment, Quzhou University, Quzhou, 324000 China; 2 Jiaxing University, Jiaxing, China; 3 Taiyu Precision Machinery (Zhejiang) Co. Ltd, 324000 Quzhou, China

**Keywords:** Dual-spindle turning and milling machine tools, SCSSA, CNN-BiLSTM hybrid network, Thermal error modeling, Engineering, Materials science, Mathematics and computing

## Abstract

Thermal error is the key factor affecting the machining accuracy of high-precision CNC machine tools. In order to solve the problem of thermal error of dual-spindle turning and milling machine tools, this study proposes an optimized CNN-BiLSTM Hybrid Network based on the SCSSA. Firstly, the thermal error experiment was carried out on the 1200MSY turning-milling double-spindle composite machine, and the temperature field data and thermal error data corresponding to the two spindles were collected. The K-means clustering-grey association coupling algorithm was used to screen the temperature-sensitive measurement points. Subsequently, the hyperparameters of the CNN-BiLSTM network were optimized with SCSSA to improve its modeling efficacy. The experimental findings indicate that: (1) SCSSA exhibits a superior convergence rate and enhanced global search capability compared to GWO and WOA algorithms during parameter optimization; (2) the proposed model achieves reductions in RMSE and MAE by 38.77% and 47.06%, respectively, when compared to benchmark models; (3) the prediction error variations are maintained within ± 4.6 μm under multiple speed conditions and ± 5 μm across various spindle types. This study provides an intelligent prediction scheme with high engineering practicality for thermal error prediction of dual-spindle turning-milling compound machine tools.

**Supplementary Information:**

The online version contains supplementary material available at 10.1038/s41598-025-27599-1.

## Introduction

The machining accuracy of CNC machine tools is affected by multiple factors, primarily including errors caused by thermal errors, geometric errors, cutting forces, and other types of errors that collectively influence machine tool machining accuracy^1^. Among these, thermal errors are the main source of precision errors in machine tool processing, accounting for 40%-70% of the total errors and being a key factor limiting the improvement of machining accuracy^2^. Therefore, researching efficient and accurate thermal error modeling methods is of great significance for improving the machining accuracy of CNC machine tools.

Currently, methods for modeling external heat error are mainly divided into two categories: heat error avoidance and data-driven heat error compensation^3,4^. Thermal error avoidance focuses on reducing the generation of thermal errors from the source by optimizing machine tool hardware design, such as improving structures, adopting low-energy components, and optimizing cooling systems. In contrast, thermal error compensation establishes thermal error models to predict and compensate for the original errors of the machine tool. Compared to thermal error avoidance, thermal error compensation offers multiple advantages, including low cost, high flexibility, and strong adaptability. However, during machine tool operation, thermal errors vary in real time according to changes in environmental and operational conditions, exhibiting highly nonlinear characteristics, numerous influencing factors, and strong coupling^5^. Therefore, thermal errors are difficult to predict. In recent years, with the development of machine learning technology, numerous scholars have made significant progress in machine tool thermal error modeling.

Li^6^ established a thermal error prediction model based on optimizing a BP neural network using an improved particle swarm optimization (IPSO) algorithm. A SOM neural network was employed to cluster temperature measurement points, and a correlation analysis method was utilized to investigate the correlation between thermal sensitive points and thermal error. The IPSO algorithm was used to optimize the initial weights and thresholds of the BP neural network. The results indicate that, compared to the GA-BP prediction model, the IPSO-BP neural network prediction model demonstrates superior modeling efficiency, robustness, and accuracy. Additionally, it can accurately predict the main spindle thermal error under different working conditions.Ali M^7^ proposed a grey prediction model optimized by cuckoo search (CS) for machine tool thermal error prediction, and by comparing it with a grey prediction model based on PSO, demonstrated the feasibility of the CS-optimized grey model.Li^8^ addresses the nonlinear time-varying characteristics of thermal errors in machine tools by proposing a BP neural network optimized with a bat algorithm (BA-BP). By employing swarm intelligence optimization, the limitations of traditional BP networks in cross-condition robustness are overcome, where the bat algorithm optimizes the initial weights and thresholds of the BP network. Experimental validation demonstrates that, compared to traditional BP networks, the BA-BP model exhibits superior stability, prediction accuracy, and robustness.Ye^9^ combined the variable selection capability of the least absolute shrinkage and selection operator (LASSO) with the efficient modeling of eXtreme Gradient Boosting (XGBoost), addressing the limitations of traditional methods under collinearity and dynamic conditions. Compared with OLS, LASSO-SVM, and RF algorithms, the prediction accuracy of thermal error improved by more than 14.5%.Zhu^10^ addresses the issue of thermal error non-linearly affecting the precision of machine tools by proposing a thermal error prediction method based on random forests and feature optimization. The model optimizes hyperparameters through grid search combined with cross-validation. By iteratively eliminating redundant features, it achieves the selection of key temperature points. Additionally, a time lag determination method based on feature importance permutation is proposed, effectively considering the lag effects between temperature changes and deformation. Experimental validation demonstrates that, compared to traditional machine learning methods, this approach offers advantages such as reduced training data requirements, faster and more intuitive parameter tuning, and higher prediction accuracy.Shi^11^ In order to decrease the thermally induced positioning error of machine tools, by proposing a Bayesian neural network (BNN)-based thermal error prediction approach. Temperature-sensitive points were selected using fuzzy c-means clustering with Dunn index optimized group numbers, effectively inhibiting multicollinearity among measuring points. Experimental validation demonstrated that the BNN model reduced maximum positioning error by 71% (from 18.2 μm to 5.14 μm) compared to BP neural networks and multiple linear regression, while maintaining superior prediction accuracy and robustness across varying working conditions. Chen et al.^12^ to address temporal lags among temperature measurement points in thermal error prediction, developed a thermal error prediction using a GRU based time series neural network with an attention mechanism. This approach leverages weighted historical temperature sequences to capture thermoelastic hysteresis effects while integrates real-time working condition data from CNC systems for thermal error prediction. Experimental validation demonstrates that the proposed model outperforms conventional RNN/LSTM methods, achieving a compensation rate exceeding 75% and reducing thermal error from 20 μm to 5 μm.

The modeling process for thermal errors in machine tools described above is largely based on temperature and thermal error data collected through experiments, with the data of temperature-sensitive points after screening being used as input for modeling. Optimization algorithms are employed to optimize parameters in order to enhance the performance of the predictive model. Although the above research has made remarkable progress, there are still many limitations: for example, insufficient consideration of the time series characteristics of thermal error leads to limited prediction accuracy. Although network models such as gated recurrent units (GRU) are used, which possess strong adaptability to data temporal sequences, GRU can only process data in temporal order and cannot utilize future information for prediction. At the same time, most of them use a single neural network model, which is difficult to capture the high nonlinearity of thermal error and the correlation between many influencing factors and high degree of coupling. In addition, most models are only validated at a fixed speed of a single spindle, lacking the operating conditions of multiple speeds in a two-spindle machine The performance evaluation restricts the engineering practicability of the model in the real-world multi-spindle collaborative machining scenario.

In order to solve these problems, this paper proposes a thermal error prediction model for dual-spindle turning and milling machine tools based on SCSSA-CNN-BiLSTM to further improve the dual-spindle turning and milling machine tools thermal error prediction effect.The convolutional neural network (CNN) component is highly effective in automatically extracting these spatial local features and sensor relationships from multiple temperature input data, which has a significant advantage over simply using flat LSTM inputs. BiLSTM is composed of forward and backward LSTM layers, and combines the characteristics of LSTM with the ability to process bidirectional data. This bidirectional structure is of great value because it enables the model to understand background information from past and future states, which is helpful in capturing the lag effects that often occur during heat treatment processes.For the screening of thermal sensitive points, the temperature rise data under the set speed of the machine tool was processed by combining K-means and gray correlation, and the temperature rise data of the temperature sensitive points of the machine tool was used as the input of the model, and then the CNN was analyzed by the SCSSA optimization algorithm-The structural parameters of the BiLSTM network model were optimized to improve the accuracy of the prediction model. In addition, a multi-angle comparative experiment is constructed for different working conditions of the same spindle and the same working condition of the double spindle to verify the thermal error modeling for the dual-spindle turning and milling machine tools proposed in this paper validity.

## Thermal error modeling method

### Convolutional neural networks

Convolutional Neural Network is a well-known and widely used algorithm in deep learning, a deep neural network that achieves deep learning of data representation through local connections, weight sharing, and hierarchical feature extraction, serving as the core tool for processing spatial data^13^. Compared with other network models, CNNs can better identify features and make use of existing features. The convolutional layer is one of the core parts of CNN, which performs convolution operations on the input data and extracts the latent features of the data. The calculation formula is as follows:

1 $${H_i}=g\left( {{W_i} \otimes {X_{i - 1}}+{b_i}} \right)$$

where:${H_i}$represents the feature quantity output by the* i* layer; ${W_i}$and ${b_i}$denote the bias and weights; $\otimes$represents the convolution operation; ${X_i}$represents the input data;* g* is the $\operatorname{Re} lu$activation function.

The main function of the pooling layer is to reduce the dimensionality of the output data of the convolutional layer, remove the redundancy of information, and speed up the calculation. At the same time, the feature has translational invariance. The calculation formula is as follows:

2 $${H_{i+1}}={P_i}\left( {{H_i}} \right)$$

Where:${P_i}$ is the pooling function,${H_i}$and ${H_{i+1}}$are the feature quantities before and after pooling, respectively.

### Bidirectional long short-term memory network

**Fig. 1:**
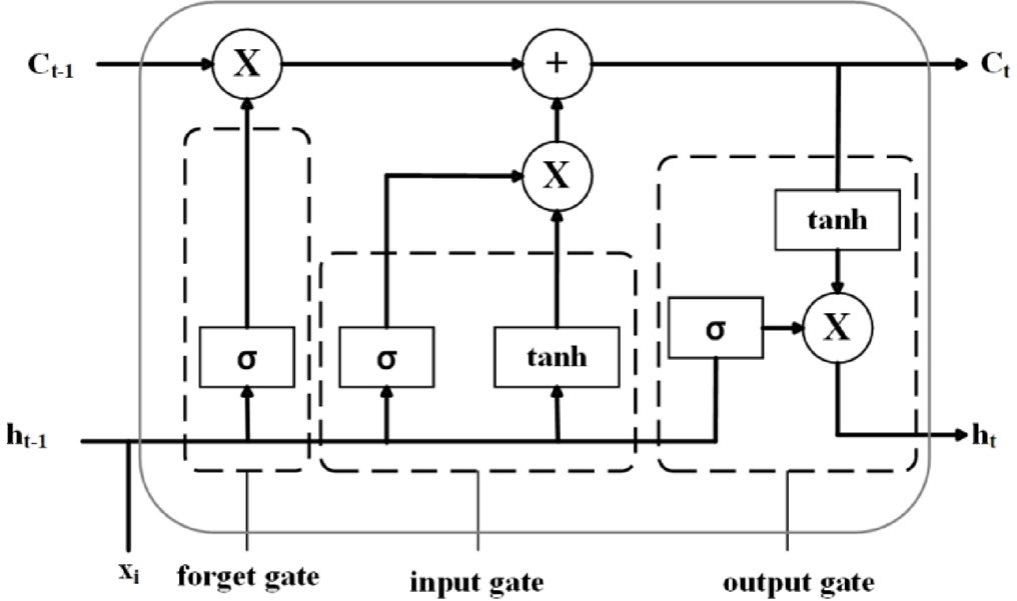
LSTM structure diagram.

Recurrent neural network (RNN) is a powerful neural network that processes sequential data, but RNN neural network has the problem of gradient vanishing. Gradient vanishing refers to the fact that in RNNs, it is difficult for RNNs to capture long-distance data dependencies as the length of the time series increases. LSTM can effectively solve the gradient vanishing problem in RNNs, especially in processing long-distance time series data, which is much better than recurrent neural networks^14^. The LSTM structure is shown in Fig. 1.

The LSTM has three new basic gating structures: the forgetting gate, the input gate, and the output gate. The formula is as follows:

3 $$\left\{ {\begin{array}{*{20}{c}} {{f_t}=\sigma \left( {{W_f} \cdot \left[ {{h_{t - 1}},{x_t}} \right]+{b_f}} \right)} \\ {{i_t}=\sigma \left( {{W_i} \cdot \left[ {{h_{t - 1}},{x_t}} \right]+{b_i}} \right)} \\ {\widetilde {{{C_t}}}=\tanh \left( {{W_c} \cdot \left[ {{h_{t - 1}},{x_t}} \right]+{b_c}} \right)} \\ {{C_t}={f_t}{C_{t - 1}}+{i_t}\widetilde {{{C_t}}}} \\ {{o_t}=\sigma \left( {{W_o} \cdot \left[ {{h_{t - 1}},{x_i}} \right]+{b_o}} \right)} \\ {{h_t}={o_t}\tanh \left( {{C_t}} \right)} \end{array}} \right.$$

Where:${f_t}$ represents the state of the forget gate at time* t* ;${i_t}$ represents the state of the input gate at time * t*; ${o_t}$represents the state of the output gate at time * t*; ${x_i}$represents the input vector at time * t*; ${h_{i - 1}}$is the output vector at time * t*; $\sigma$ is the $sigmoid$activation function; * W*is the weight matrix; * b* is the bias; $\widetilde {{{C_t}}}$is the candidate cell state; and ${C_t}$is the new cell state.

**Fig. 2:**
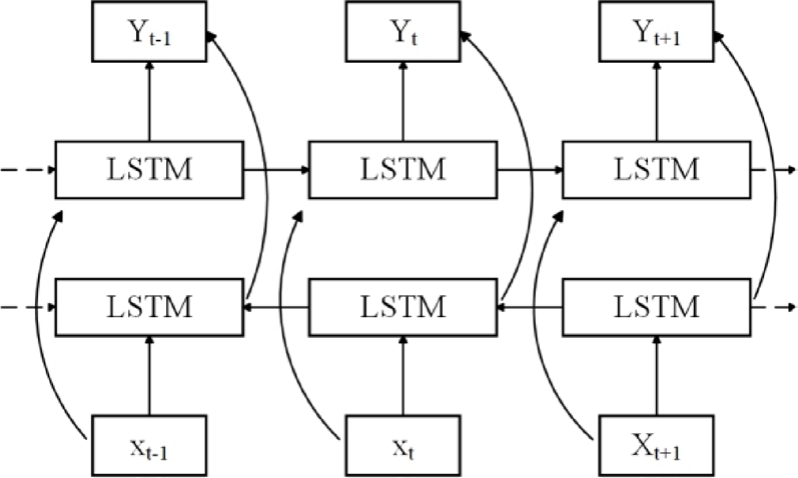
Structure of BiLSTM.

The BiLSTM model consists of a forward-facing LSTM layer and a backward LSTM layer, and the two LSTM layers are trained from the front and back of the data, so that the sequence can be processed in both directions, and the internal relationship between the current data and the data in the past and future tense can be further explored. It can improve data utilization and model prediction accuracy. The BiLSTM structure is shown in Fig. 2.

### Sparrow search algorithm that fuses sine and cosine and cauchy variation

#### Sparrow search algorithm combining sine-cosine and cauchy mutation

Sparrow Search Algorithm (SSA)^15^ is a meta-heuristic optimization algorithm based on the foraging and anti-predation behaviors of sparrow groups. SSA achieves global optimization in the parameter space through the synergistic interaction of three functional categories: discoverers, scroungers and investigation early warning mechanism. However, SSA has the problems of insufficient ability, loss of population diversity, and easy to fall into local extremes in the later stage of optimization. Therefore, this paper introduces a Sparrow Search Algorithm Combining Sine-Cosine and Cauchy Mutation(SCSSA).

##### Refractive inverse learning strategy

SCSSA uses a dimension-by-dimension inverse learning strategy for small hole imaging. By synergizing the inverse learning strategy of small hole imaging and dimension-by-dimension reverse learning, the algorithm can get rid of the local extremum^16^ by performing a dimension-by-dimension inverse solution for a feasible solution. This strategy takes the top individuals in fitness ranking as the initial population, which reduces the risk of the algorithm falling into the local optimal solution. The initialization formula for the sparrow population is:

4 $$x_{{i,j}}^{*}=\frac{{{l_j}+{u_j}}}{2}+\frac{{{l_j}+{u_j}}}{{2k}} - \frac{{{x_{i,j}}}}{k}$$

Where:${x_{_{{i,j}}}}$ represents the position of the* i* sparrow in the* j* dimensional space, $x_{{i,j}}^{*}$ represents the reverse refraction solution of ${x_{i,j}}$; ${l_j}$ and ${u_j}$ are respectively the minimum and maximum values of the * j* dimension in the search space; * k*as a regulatory factor

##### Sine-cosine algorithm

In the sparrow search algorithm, the spatial distribution of food sources directly affects the search direction and collaboration efficiency of individuals in the population, and the uneven distribution of food sources will lead to the producers falling into the local optimal solution. The homogeneous migration of scroungers leads to a decrease in population diversity and increases the likelihood of falling into local extremes. In view of this phenomenon, the sine-cosine algorithm (SCA)^17^ is embedded in the producers position update. SCA uses the oscillation variation characteristics of the sine and cosine model to act on the position of the discoverer, and performs global and local optimization to obtain the global optimal value. Get a new producers location update formula as:

5 $$X_{{t+1}}^{{i,j}}=\left\{ {\begin{array}{*{20}{c}} {\omega \cdot X_{t}^{{i,j}}+r_{1}^{\prime } \cdot \sin {r_2} \cdot \left| {{r_3} \cdot {X_{best}}} \right|,}&{{R_2}<ST} \\ {\omega \cdot X_{t}^{{i,j}}+r_{1}^{\prime } \cdot \cos {r_2} \cdot \left| {{r_3} \cdot {X_{best}}} \right|,}&{{R_2} \geqslant ST} \end{array}} \right.$$

Where: ${r_2} \in \left[ {0,2\pi } \right]$is a random number that determines the movement distance of the sparrow; ${r_3} \in \left[ {0,2\pi } \right]$ is a random number used to control the influence of the optimal individual on the subsequent position of the sparrow; $r_{1}^{\prime }$ is a nonlinear decreasing search factor; $\omega$is a nonlinear weight factor.

##### Cauchy mutation strategy

In the process of foraging for sparrows, the competition between individuals for food often makes scroungers become discoverers, resulting in the decline of population diversity. To prevent the algorithm from falling into local optima, the Cauchy mutation strategy is introduced in the scrounger update formula to enhance global optimization capabilities. Below is the updated follower position formula:

6 $$X_{t}^{{i,j}}={X_{best}}\left( t \right)+cauchy\left( {0,1} \right) \cdot {X_{best}}\left( t \right)$$

Where:$cauchy\left( {0,1} \right)$ represents the standard Cauchy distribution function. The Cauchy distribution and the normal distribution are both continuous real-valued probability distributions, but the Cauchy distribution has lower probability near the origin, slower tail exponential decay with higher probability, and a significantly higher probability of extreme values compared to the normal distribution. Therefore, by applying Cauchy variation to perturb the position of the sparrow, the search range of the sparrow search algorithm can be expanded, thereby enhancing the algorithm’s potential to escape local optimal solutions^18^.

##### Investigation early warning mechanism

Considering the safety of the sparrow population and the ability to obtain food, the sparrow will select 10%~20% of the individuals from the population for reconnaissance and vigilance, and the location will be updated as follows:

7 $$X_{{i,j}}^{{t+1}}=\left\{ {\begin{array}{*{20}{c}} {X_{{best}}^{t}+\beta \left| {X_{{i,j}}^{t} - X_{{best}}^{t}} \right|,}&{{f_i}>{f_g}} \\ {x_{{i,j}}^{t}+k \cdot \left( {\frac{{\left| {X_{{id}}^{t} - X_{{worst}}^{t}} \right|}}{{\left( {{f_i} - {f_w}} \right)+\varepsilon }}} \right),}&{{f_i}={f_g}} \end{array}} \right.$$

Where:$\beta$ is a random number generated from the standard normal distribution;* k* is a random number between $\left[ { - 1,1} \right]$; $\varepsilon$ is a very small constant, the purpose of which is to avoid the inability to update the position when the denominator is 0; ${f_i}$ represents the fitness of the * i* th sparrow, ${f_{_{g}}}$ and ${f_w}$ are respectively the best and worst fitness values of the current sparrow population.

In summary, the SCSSA algorithm integrates the adaptive adjustment mechanism of the SCA, the initialization strategy of the refractive reverse learning, and the global disturbance ability of the Cauchy mutation. By doing so, it significantly improves the convergence speed, accuracy, and robustness while maintaining the original framework of SSA, providing a more effective solution for complex optimization problems.

### Screening of temperature sensitive points

In order to improve the efficiency of model modeling and reduce the amount of calculation, K-means and grey correlation analysis are used to screen the temperature sensitive points of the machine tool spindle. The basic steps are: K-means divides the data into K groups in advance, randomly selects K objects as the initial clustering center, and then The Euclidean distance from each sequence to the center of mass is calculated. According to the similarity between the data and the clustering center, the position of the clustering center is continuously updated, and the Sum of Squared Error (SSE) of the cluster is continuously reduced ), when the SSE no longer changes or the objective function converges, the clustering ends to obtain the final result^19^. In the K-means clustering algorithm, determining a suitable K value (i.e., the number of clusters) is a key problem. In this paper, the Gap Statistical method is used to determine the indicators. The following is the definition of Gap:

8 $$Gap\left( k \right)={E_n}^{ * }\left( {\log \left( {{D_K}} \right)} \right) - \log \left( {{D_k}} \right)$$

Where:$\log \left( {{D_k}} \right)$represents the dispersion of observed data clustering; ${E_n}^{*}\left( {\log \left( {{D_K}} \right)} \right)$is the expected value of $\log \left( {{D_k}} \right)$. The basic process of the algorithm is first to generate uniformly distributed random samples in the region where the samples are located, with the same number as the original samples, and to perform K-means clustering on this random sample multiple times to obtain multiple ${D_k}$ values. The logarithms of these multiple ${D_k}$ values are taken and averaged to obtain an approximate value of ${E_n}^{*}\left( {\log \left( {{D_K}} \right)} \right)$. When the interval statistic ${E_n}^{*}\left( {\log \left( {{D_K}} \right)} \right)$ achieves its maximum value, the corresponding * k* value is the opti.mal number of clusters^20^.

Grey relational analysis determines the relationship between temperature rise data and thermal error by calculating their correlation. In the K classes obtained from K-means clustering, the temperature measurement point with the highest correlation in the temperature rise data is identified as the temperature sensitive point for that class, thereby screening out the temperature sensitive points for predicting the main axis thermal error.The grey correlation analysis is as follows:

9 $$\zeta \left( {y,{x_j}} \right)=\frac{1}{n}\sum\limits_{{t=1}}^{n} {\frac{{{{\hbox{min} }_j}{{\hbox{min} }_t}\left| {y\left( t \right) - {x_j}\left( t \right)} \right|+\rho {{\hbox{max} }_j}{{\hbox{max} }_t}\left| {y\left( t \right) - {x_j}\left( t \right)} \right|}}{{\left| {y\left( t \right) - {x_j}\left( t \right)} \right|+\rho {{\hbox{max} }_j}{{\hbox{max} }_t}\left| {y\left( t \right) - {x_j}\left( t \right)} \right|}}}$$

Where:$\rho =0.5$is the resolution coefficient; $y\left( t \right)$ is the target sequence; ${x_j}\left( t \right)$ is the comparison sequence.

### Experimental data processing

To enhance the training efficiency and generalization performance of the model, the input data of the model is preprocessed. At the same time, to preserve the integrity of the original data and ensure the correct scaling of the model input, only normalization is used to preprocess the original data. Normalization converts the original temperature data into a range of [-1, 1]. The formula for normalization is as follows:

10 $${X_{norm}}=\frac{{X - {X_{\hbox{min} }}}}{{{X_{\hbox{max} }} - {X_{\hbox{min} }}}}$$

Where: *X* represents the original temperature data;${X_{\hbox{min} }}$and${X_{\hbox{max} }}$ represent the minimum and maximum values of the dataset, respectively༛${X_{norm}}$ represents the normalized value (range).

## Thermal error experiment of machine tool spindle

### Design of experiments

**Fig. 3:**
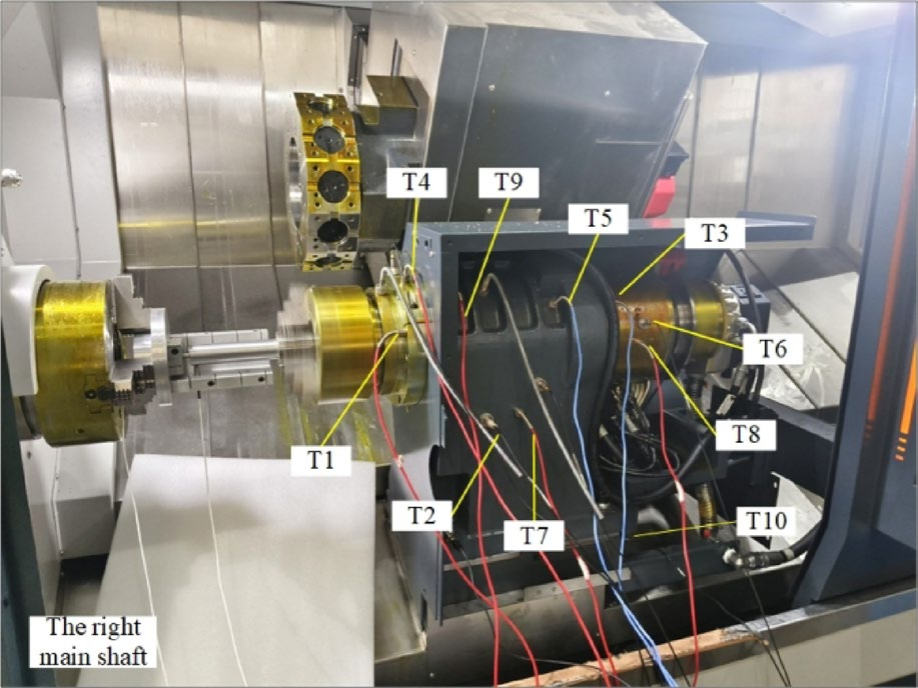
The distribution of the main shaft sensors on the right side of the machine tool. T1, T3, T4, T6, T8 measure the temperature change at various points of the spindle, T2, T5, T7, T9, T10 measure the temperature change throughout the headstock, and T11 is the ambient temperature.

To verify the feasibility of the proposed SCSSA-CNN-BiLSTM thermal error prediction model, experiments were conducted on a dual-spindle turning-milling machine to obtain temperature field data and thermal error data for the left and right spindles. In the experiment, two thermal error sensors were mounted on the workbench with the workpiece to measure the axial and radial thermal displacement data of the spindles. Eleven PT100 platinum resistance temperature sensors were arranged at the core parts of the machine spindles to measure the temperature rise of the machine spindles. It was found during the experiment that the radial thermal error values of the spindles were within ± 0.2 μm, while the axial thermal error of the spindles was more pronounced. Therefore, this paper selects the axial thermal error of the spindles as the primary research object. The thermal error sensor and temperature sensor arrangement are shown in Fig. 3.

In the actual machining process, the machine spindle will show a variety of working states. In order to ensure that the thermal error prediction model has generalization ability and accuracy, experiments on the right spindle at different speeds (spindle idling) were designed to target different working conditions. The details are shown in Table 1.

**Table 1:** Design of working conditions for different rotational speeds of the machine tool.

Grouping	Rotational speed (*r*/min)
1	2000
2	5000
3	8000
4	10,000

**Fig. 4:**
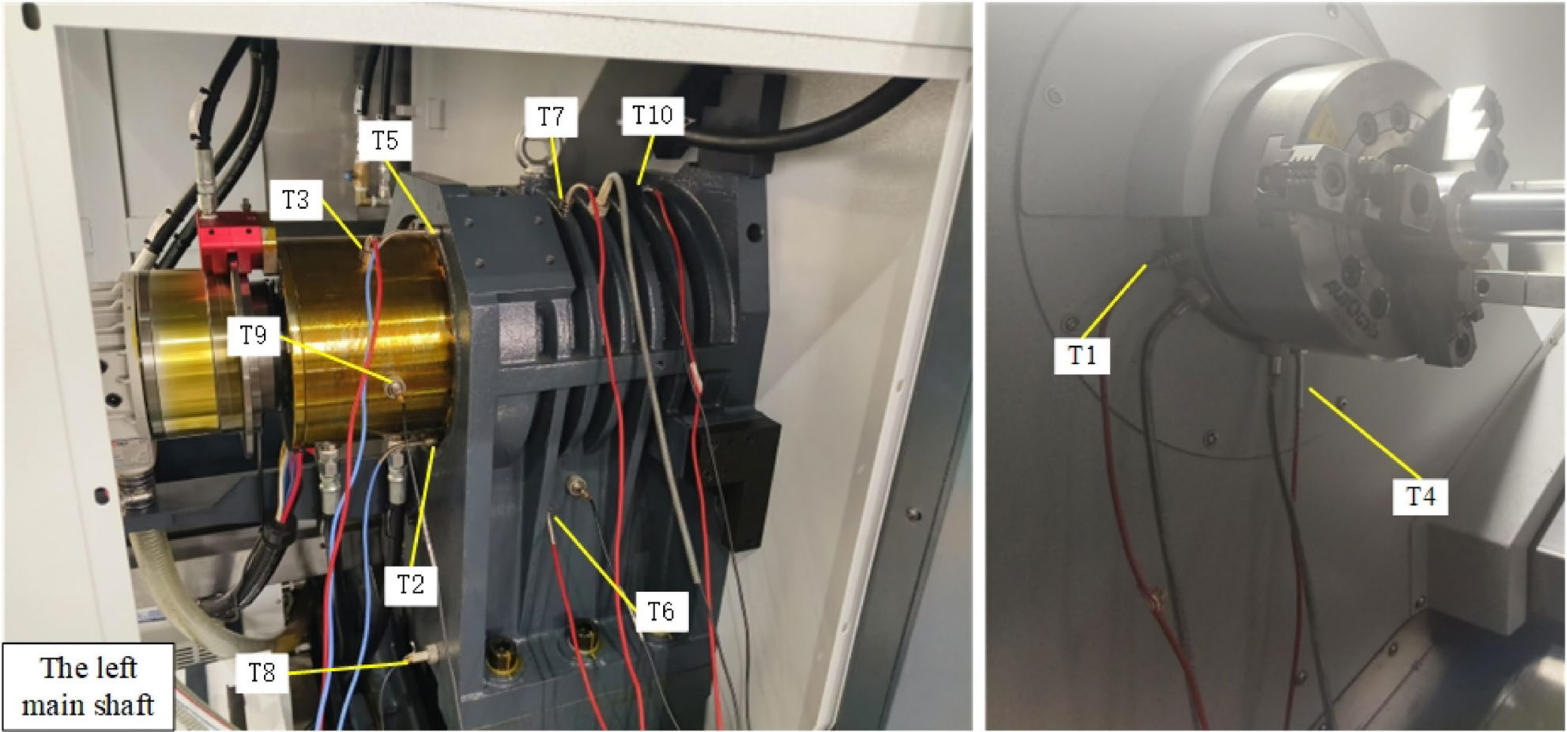
Temperature distribution points of the main spindle on the left side of the machine tool.

In order to verify the applicability of the dual-spindle thermal error prediction model to the dual-spindle structure, the left-hand spindle 8000r/min corresponding to the 8000r/min condition of the right-hand spindle was designed Thermal error experiments to test the predictive performance of the model on different spindles. The arrangement of the left main shaft thermal error sensor and temperature sensor is shown in Fig. 4.

### Experimental data presentation

**Fig. 5:**
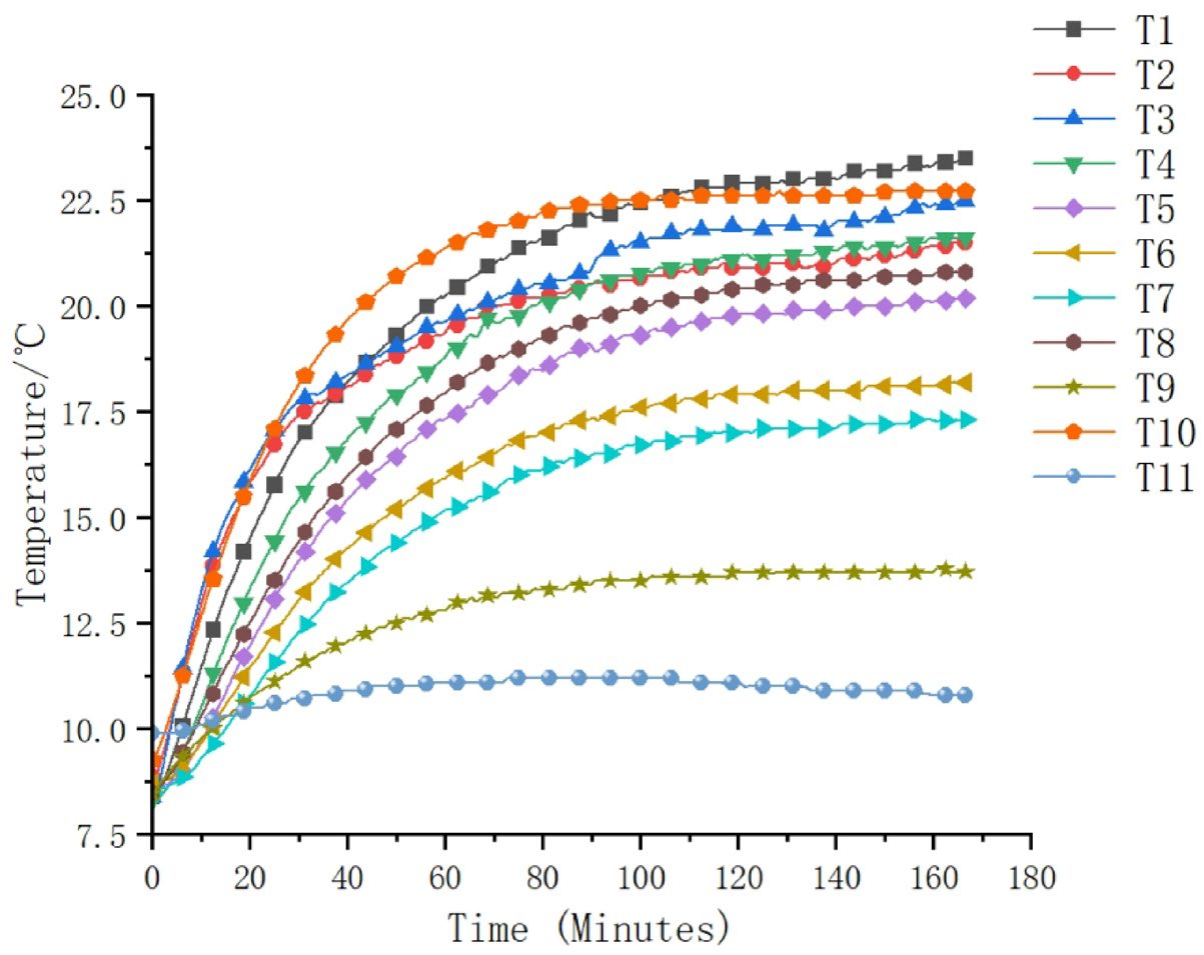
Temperature rise sequence diagram of the main shaft at different measurement points at 8000 r/min.

**Fig. 6:**
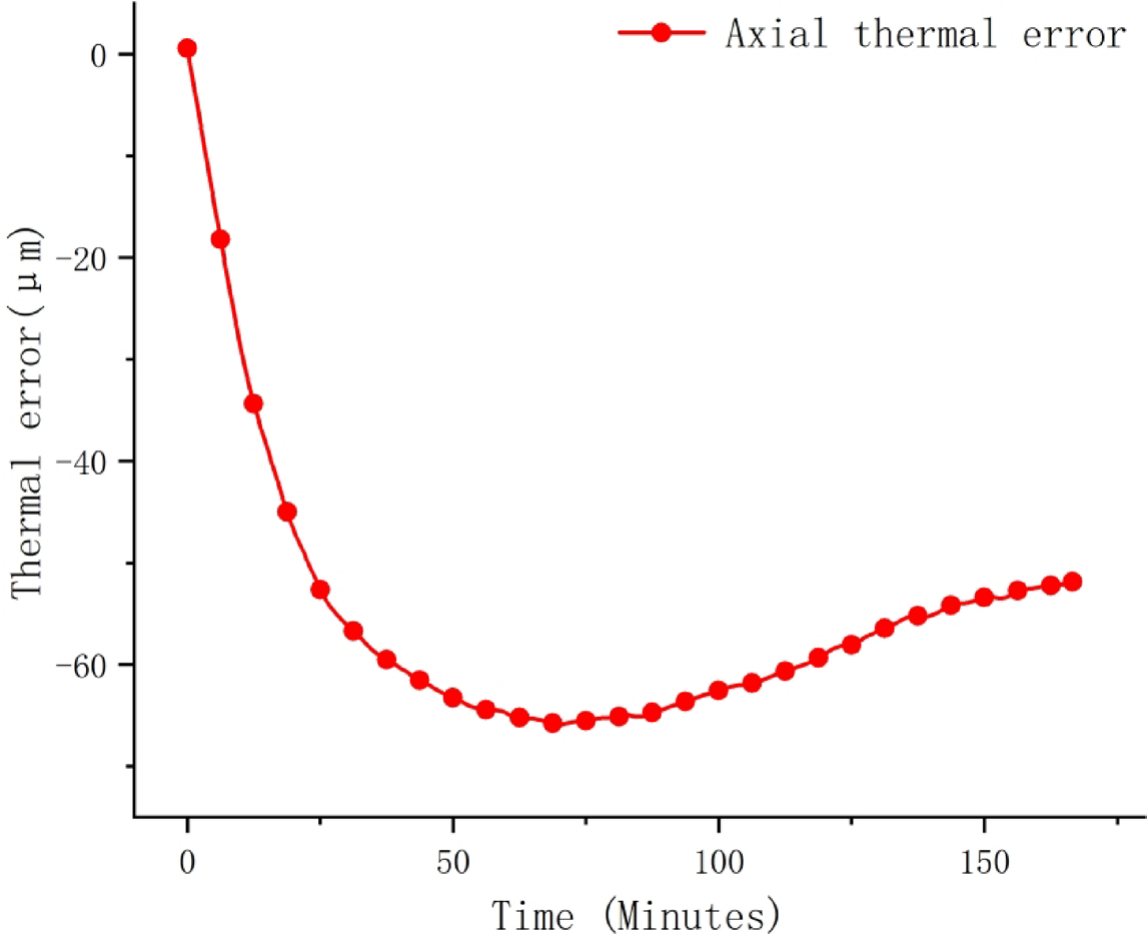
Thermal error time sequence diagram of the main spindle operating at 8000 r/min.

To address the issues of data set sufficiency and model generalization ability, a total of 2000 data samples were collected during the experiment. Each sample contained a multi-dimensional input vector (temperature readings from 11 sensors) and the corresponding axial thermal error measurement value. The data was collected every 5 s and was continuously run for several hours at different rotational speeds (2000 rpm, 5000 rpm, 8000 rpm, and 10000 rpm) to capture transient and steady-state thermal behaviors. The data set was divided using a systematic approach: one sample out of every 10 was selected as the test set, resulting in 200 test samples and 1800 training samples. Figures 5 and 6 are the timing diagrams of the 11 temperature rise data and the axial thermal error of the spindle at 8000 r/min, respectively.

## Establishment of thermal error model

### Critical temperature sensitive points are determined

During the experimental design phase, in order to ensure that the temperature rise data of various parts of the machine tool spindle could be collected, 11 temperature sensors were placed at different locations on the spindle. However, too many temperature measurement points may cause the problem of data collinearity, and excessive data collinearity will lead to an increase in the complexity of thermal error modeling calculations and a decrease in modeling efficiency.

**Fig. 7:**
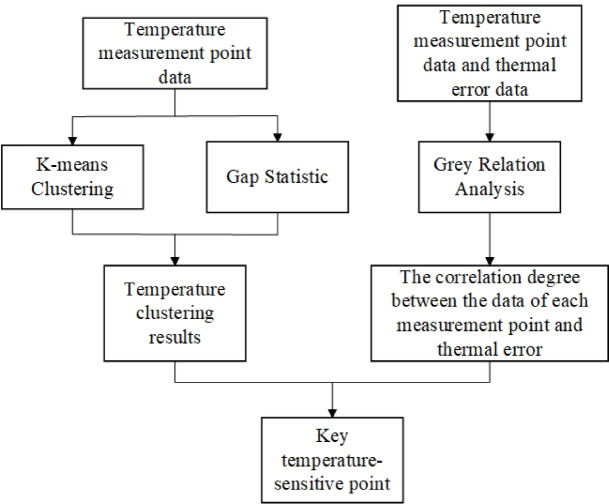
K-means clustering analysis and grey correlation analysis flowchart.

Through K-means clustering analysis and grey correlation analysis, the classification and correlation degree of the 11 temperature measurement points were calculated respectively. The specific flowcharts of K-means clustering analysis and grey correlation analysis are shown in Fig. 7.

The grey correlation analysis shows that under the condition where the right main shaft rotates at a speed of 8000 r/min, the calculation results of the grey correlation degrees at 11 temperature points are presented in Table 2.

**Table 2:** Grey relational grades of temperature Points.

Temperature measurement point	Gray correlation coefficient
T1	0.8447
T2	0.8533
T3	0.8404
T4	0.8448
T5	0.8321
T6	0.8341
T7	0.8310
T8	0.8337
T9	0.8270
T10	0.8704
T11	0.7680

The final clustering results and the selected key temperature-sensitive points are shown in Table 3. The temperature-sensitive points under five different operating conditions are presented in Table 4.

**Table 3:** Key temperature-sensitive point.

Grouping	K-means clustering grouping	Temperature sensitive points
1	T1, T2, T3, T10	T10
2	T4, T5, T8	T4
**3**	T6, T7	T6
**4**	T9, T11	T9

**Table 4:** Temperature-sensitive points under different working conditions.

Different working conditions	The right spindle is 2000r/min	The right spindle is 5000r/min	The right spindle is 8000r/min	The right spindle is 10000r/min	The left spindle is 8000r/min
Temperature sensitive points	T9, T10, T11	T2, T3, T9, T10	T4, T6, T9, T10	T6, T8, T9, T10	T6, T9, T10

### Design and comparison of CNN-BiLSTM hybrid network model architecture

To verify the superiority of the CNN-BiLSTM architecture, we conducted preliminary experiments on different architectures and evaluated the performance of several models on the dataset, including: CNN, LSTM, BP, and CNN-BiLSTM.The results are summarized briefly as shown in Table 5, and the predictions are explained based on the operating conditions of the operation at a rotational speed of 8000 revolutions per minute on the right main axis.

**Table 5:** Comparison of different models.

Model architecture	BP	LSTM	CNN	CNN-BiLSTM
RMSE (µm)	1.9759	1.655	1.5795	1.1315
MAE(µm)	1.4127	1.3575	1.2494	99.1637%
R²	97.45%	98.2109%	98.3707%	0.89385

From the results, our CNN-BiLSTM model performed the best, indicating that it has a strong predictive ability. Therefore, we chose the CNN-BiLSTM architecture as the base model for our thermal error modeling problem.

**Fig. 8:**
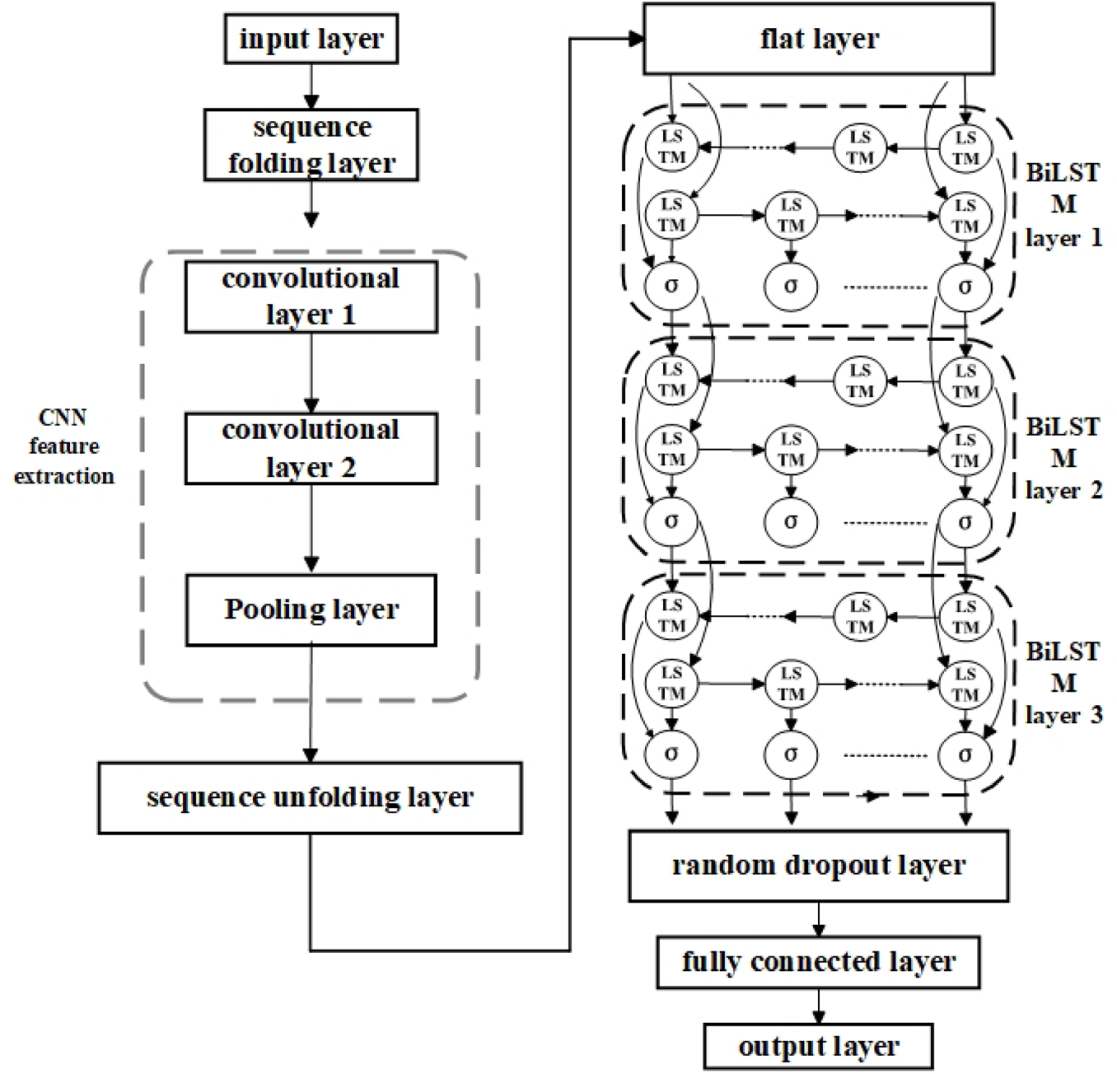
CNN-BiLSTM hybrid network model structure.

(2) The sequence folding layer is used to reconstruct the dimension of the input data to adapt to the requirements of convolution operation; The CNN feature extraction module consists of two layers of convolutional layer and one layer of pooling layer, and the size of the first layer of convolutional kernel is set to [2,1], The second layer convolution kernel size is [1,1], and the number of convolution kernels is 10, and the step size is1. Convolutional layers are used to capture the local spatial correlation of temperature data. The pooling layer adopts the maximum pooling layer method, and the [1 × 5] window is selected for feature compression, and the dimensionality reduction process is realized while retaining the salient features. Time series feature extraction module: After the data format is converted through the sequence expansion layer and the flattening layer, the time series network composed of three layers of BiLSTM stacking is input. Each BiLSTM layer is followed by Dropout layer with 0.3 probability of randomly discarding input data to enhance the generalization ability of the model; Regression output layer: The high-order spatiotemporal features are mapped to the target dimension through the fully connected layer, and the predicted value of the axial thermal error is finally output. Figure 8 shows the structure of the CNN-BiLSTM network model.

###  SCSSA-optimized CNN-BiLSTM prediction model

The hyperparameters in the network structure have a significant impact on the prediction accuracy and model fitting ability to ensure the best prediction results. The specific steps of SCSSA optimization are as follows:

Step 1: Set the sparrow population size to 30, the number of iterations to 50, and the proportion of discoverers to 30%. The search range for the number of hidden neurons in the three BiLSTM layers is set to [5,200], [5,100], and [5,100], respectively. The search range for the initial learning rate is set to [0.0001,0.01], and the search range for the L2 regularization coefficient is set to [0.0001,0.1].

Step 2: Improve the initialization of the population according to the refractive inverse learning strategy.

Step 3: Calculate the fitness of sparrow individuals according to the Root Mean Square Error (RMSE), record the position and fitness of the optimal individuals and rank them.

Step 4: Iteratively update the producers position and its fitness according to Eq. 5.

Step 5: Iteratively update the scroungers position and its fitness according to Eq. 6.

Step 6: Iteratively update the location of the sentinel and its fitness according to Eq. 7.

Step 7: When the termination iteration condition is satisfied, the optimal hyperparameters of the network are obtained. Otherwise, go back to the third step and iterate in a loop.

Step 8: After the iteration, the number of hidden layer neurons, the initial learning rate and the optimal combination of L2 regularization coefficient of the three BiLSTM layers optimized by SCSSA are input into CNN-BiLSTM network for prediction.

**Fig. 9:**
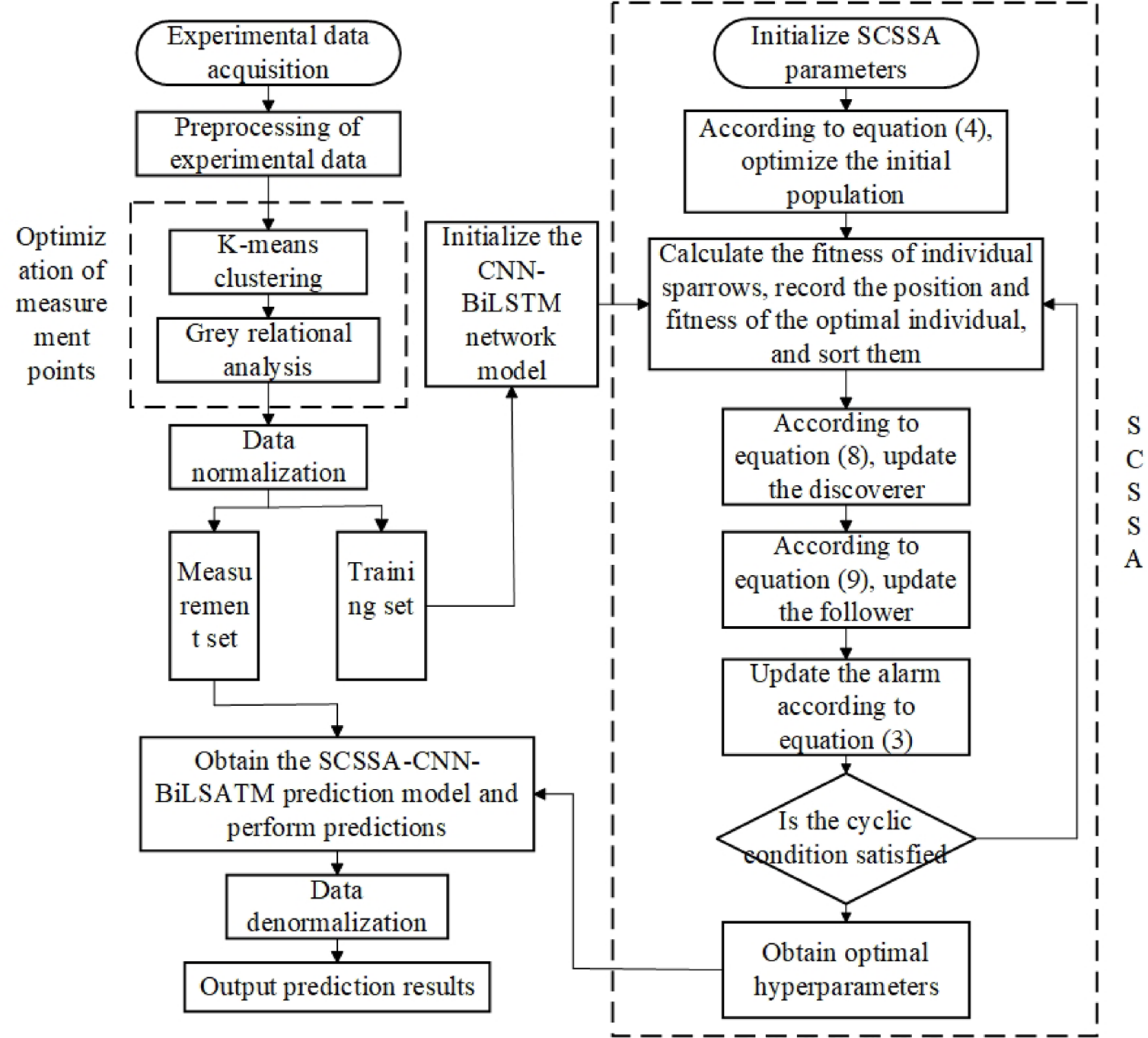
The overall optimization process of the SCSSA-CNN-BiLSTM prediction model.

The overall optimization process of the SCSSA-CNN-BiLSTM prediction model is shown in Fig. 9.

###  Performance verification and parameter optimization results of SCSSA optimization algorithm

**Fig. 10:**
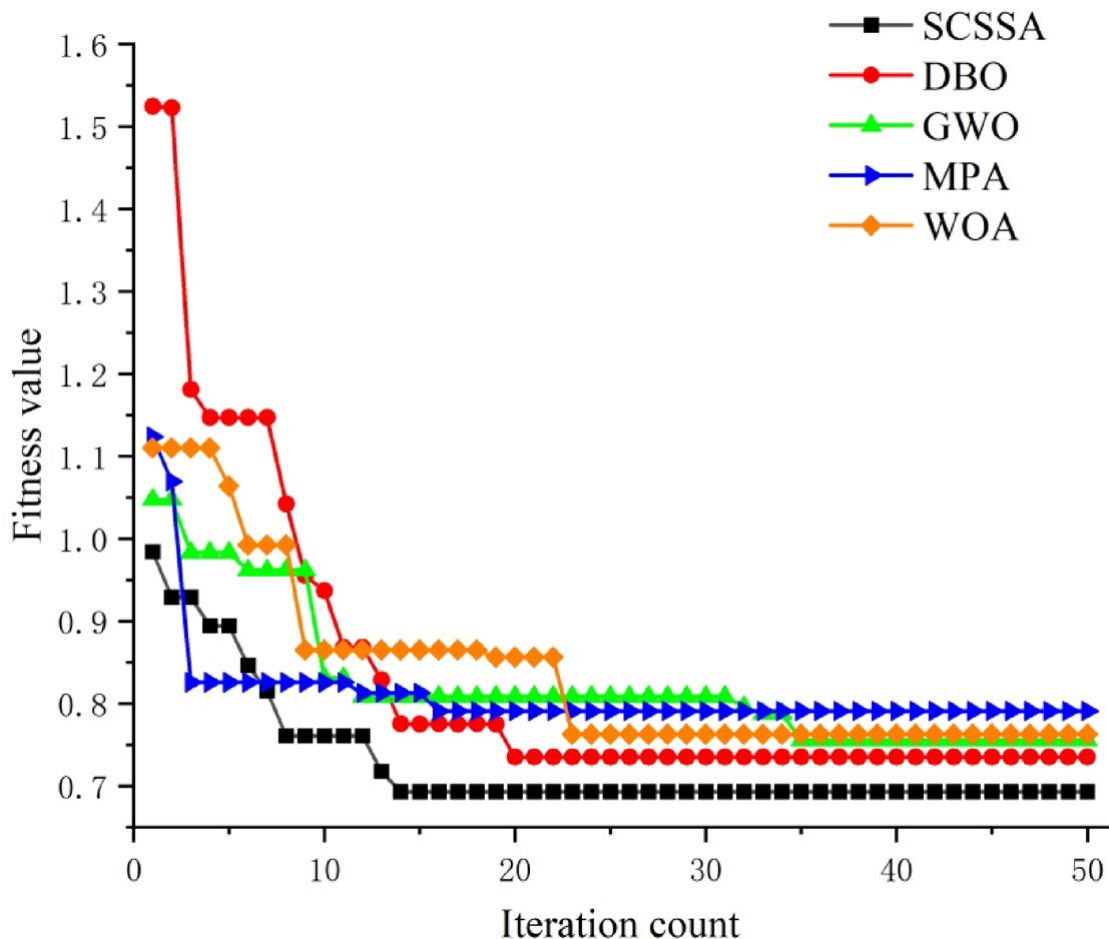
Five types of algorithm iterative change curves.

To test the performance of the SCSSA algorithm, a comparison was conducted using commonly employed algorithms: the Grey Wolf Optimizer (GWO)^21^, the Whale Optimization Algorithm (WOA)^22^, the Ocean Prey Algorithm (MPA)^23^, and the Dung Beetle Optimizer (DBO)^24^. It is applied to the CNN-BiLSTM network model for comparison and evaluation, and the number of populations and iterations in the algorithm is set to be consistent, and the experimental input data are consistent to ensure the reliability of the experimental results. Under the condition that the spindle speed is 8000r/min, the fitness values of the five optimization algorithms change in the iterative process as shown in Fig. 10.

Experimental results show that the five optimization algorithms have completed convergence within 35 iterations, and the SCSSA has a significant advantage, ranking 14thThe generation completes the convergence, and the convergence speed is the fastest. In terms of the final convergence value, the fitness value of SCSSA after convergence is 0.69267, which is the best among the five algorithms. Observing the iterative process, it is found that the GWO and WOA algorithms will experience a long period of fitness stagnation during the iteration process, while SCSSA does not have a long-term stagnation of fitness values. Among the five algorithms, the SCSSA algorithm exhibits the lowest convergence value, the fastest convergence speed and less fitness value stagnation when optimizing the hyperparameters, which is very important for CNN-BiLSTM The optimization ability of network hyperparameters is more prominent, and it has certain advantages compared with other algorithms. The hyperparameter search results of the SCSSA optimization algorithm are shown in Table 6.

**Table 6:** Optimization results of hyperparameters.

Optimization parameters	BiLSTM layer 1	BiLSTM layer 2	BiLSTM layer 3	Initial learning rate	L2 regularization coefficient
Optimize the results	200	100	54	0.0025364	0.0001

## Model prediction results and validation of the thermal error prediction model

### Comparison and analysis of prediction results with different algorithms

**Fig. 11:**
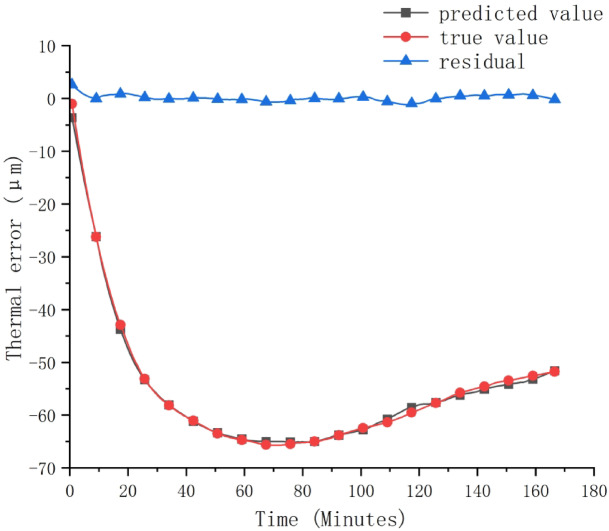
Comparison between model predicted values and actual values.

The hyperparameter results after SCSSA algorithm optimization are input into the CNN-BiLSTM hybrid network model, and the working condition that the shaft speed is 8000r/min on the right spindle is predicted The prediction results of the test set are compared with the real values, and the comparison results are shown in Fig. 11.

In order to verify the superiority of the SCSSA-CNN-BiLSTM prediction model used in this paper. A comparative analysis of thermal error prediction using BP, CNN, and CNN-BiLSTM models, and the hyperparameters of the three models were manually adjusted. The input data is consistent with the SCSSA-CNN-BiLSTM prediction model. Figure 11 illustrates the prediction results. Table 5 shows the prediction performance of each model.

**Fig. 12:**
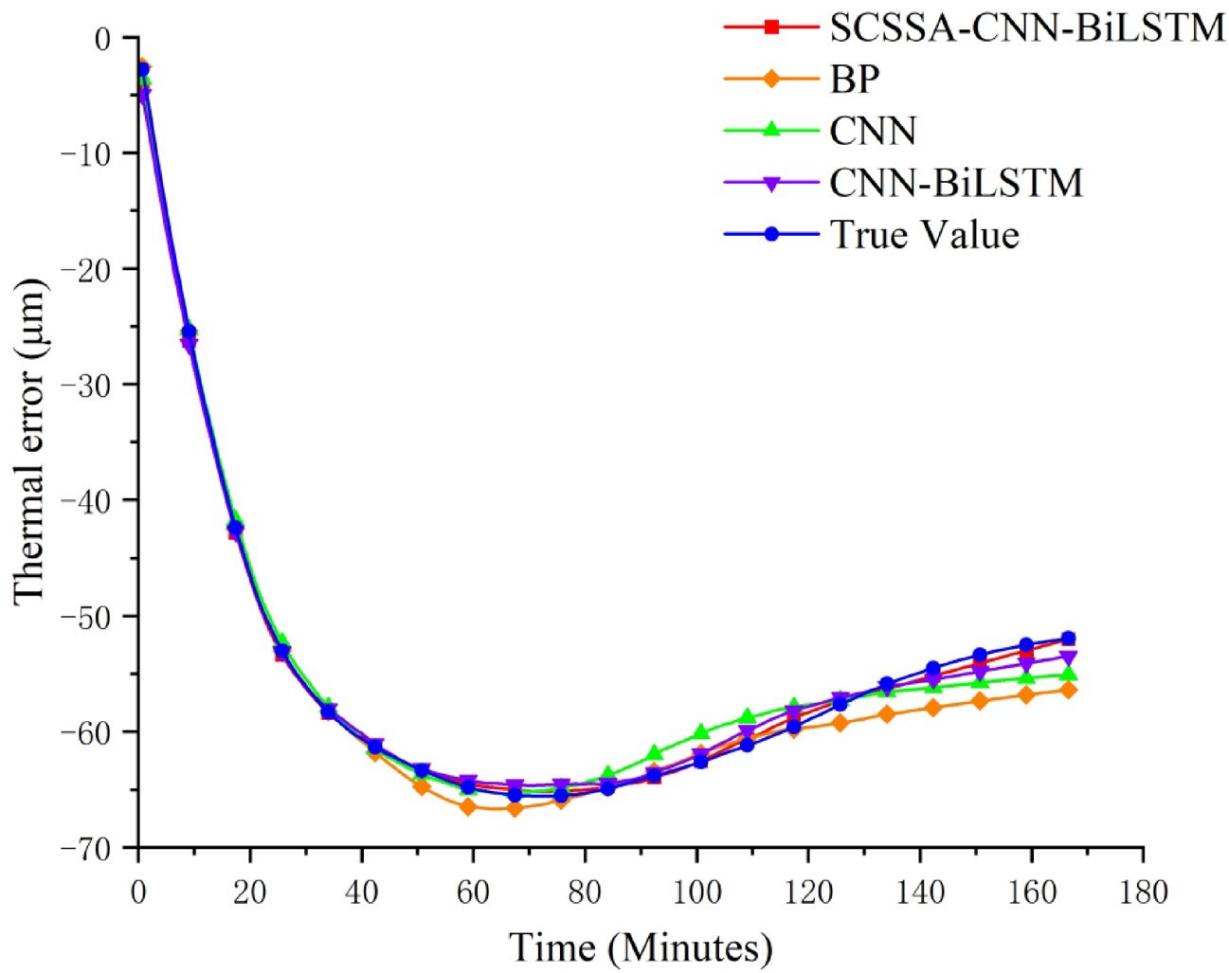
Comparison of prediction results from different models.

**Table 7:** A comparison of prediction performance across different Models.

Evaluation indicators	SCSSA-CNN-BiLSTM	BP	CNN	CNN-BiLSTM
RMSE	0.69268	1.9759	1.5795	1.1315
*R* ^2^	99.687%	97.45%	98.3707%	99.1637%
MAE	0.47319	1.4127	1.2494	0.89385

Figure 12 provides an intuitive comparison of the thermal error prediction curves of different models. Among them, the SCSSA-CNN-BiLSTM prediction model has the highest degree of coincidence with the actual thermal error values. When comparing the prediction data of the three models, the prediction error fluctuation of the SCSSA-CNN-BiLSTM model is smaller, and it has better stability in data fitting.From Table 7 shows that compared with the three contrast models, the root mean square error (RMSE) of the SCSSA-CNN-BiLSTM prediction model has decreased by 64.92%, 56.15%, and 38.77% respectively; the mean absolute error (MAE) has decreased by 66.50%, 62.11%, and 47.06% respectively, indicating that the prediction results are stable and have fewer extreme values.

This is not only a statistical improvement, but also very important in the context of industrial precision processing. Firstly, the halving of MAE indicates a significant improvement in the average prediction deviation, greatly enhancing the daily stability and consistency of processing accuracy. Secondly, the significant reduction in RMSE indicates that the model is more effective in suppressing large prediction deviations, directly helping to reduce product defects and scrap rates caused by outliers. The magnitude of this error reduction is undoubtedly sufficient to significantly improve production accuracy. The compensation model supported by these increased prediction accuracies can enhance the capabilities of the machine tool, enabling the machine to always meet more stringent tolerance requirements, which is crucial for manufacturing high-value components in industries such as aerospace, automotive, and medical equipment.

### Prediction results and comparative analysis of different working conditions

**Fig. 13:**
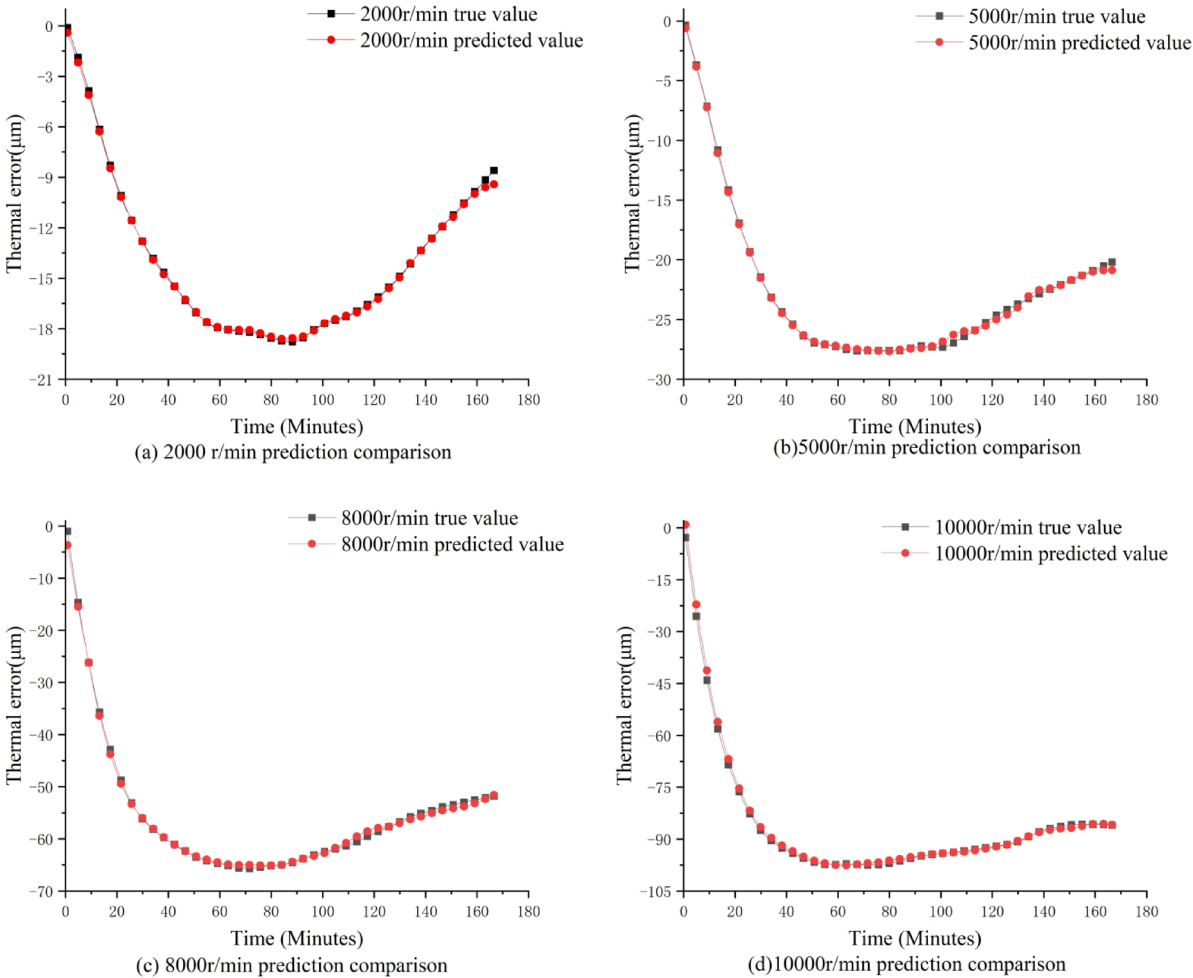
Comparison of predictions under four different rotational speed conditions.

**Fig. 14:**
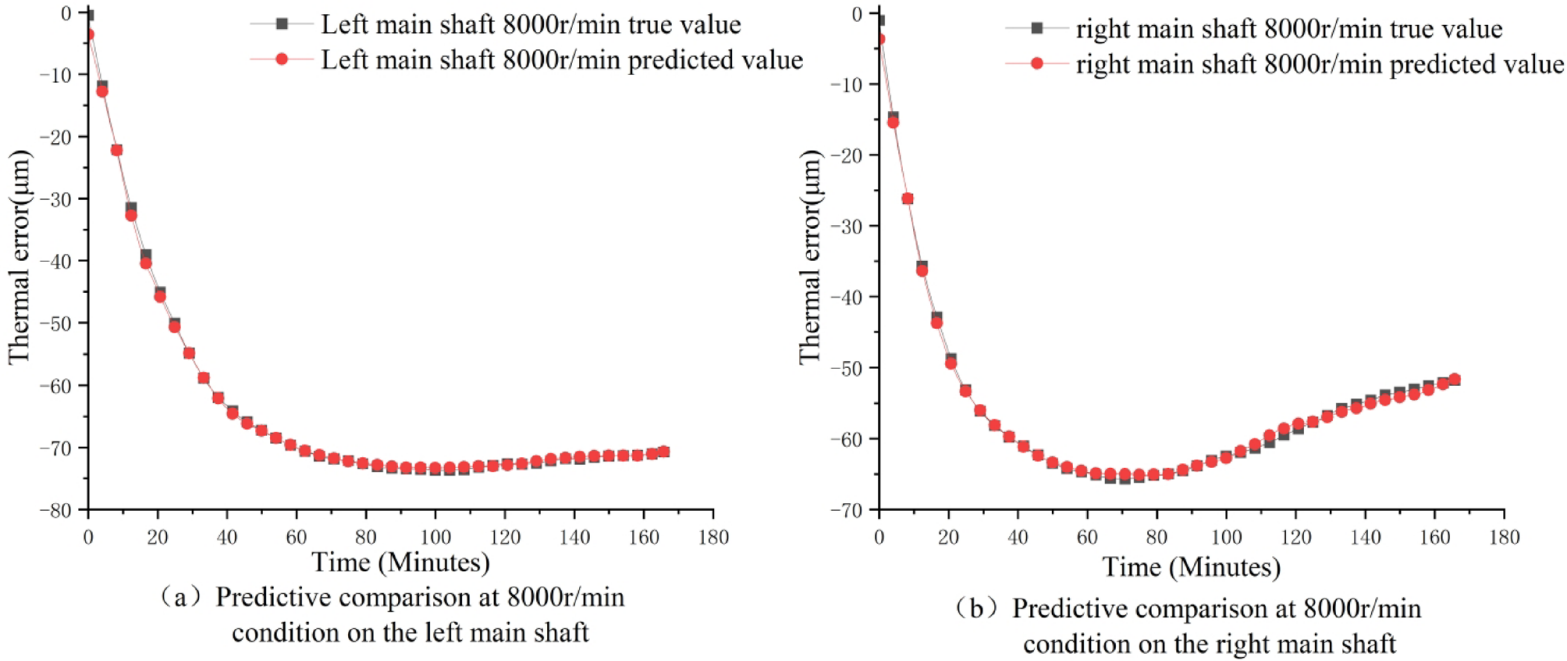
Comparison of dual-axle model predictions.

In order to determine the generalization ability of the SCSSA-CNN-BiLSTM prediction model under different rotational speeds and different spindle working conditions, the optimized hyperparameters of the SCSSA algorithm under four different rotational speed conditions (2000/5000/8000/10000 r/min) were input into the CNN-BiLSTM hybrid network of the same spindle for thermal error prediction; at the same time, to verify the adaptability of the model to different spindles, the optimized hyperparameters of SCSSA under the 8000 r/min working condition of the right spindle were input into the CNN-BiLSTM network of the other spindle for prediction effect verification. During this process, all input temperature data were preprocessed uniformly (as described in Sect. 2.5), converting the original temperature values to the range of [-1, 1]. This effectively eliminated the differences in absolute temperature values and distribution ranges caused by individual spindle differences and different rotational speeds, enabling the model to focus on learning the mapping relationship between temperature and thermal error rather than the unique characteristics of specific spindles or specific rotational speeds. This is the core prerequisite for this unified model architecture to be able to adapt to multiple rotational speeds and generalize to different spindles. The comparison results of the predicted values and the true values are shown in Figs. 13 and 14. The results indicate that the prediction results of the SCSSA-CNN-BiLSTM model under different rotational speed conditions and different spindles are all good, effectively supporting the precise prediction and compensation of thermal errors in the actual processing of the dual-spindle machine tool.

From Figs. 13 and 14, it can be seen that the consistently high prediction accuracy demonstrated at multiple speeds and between two different spindles highlights the inherent robustness of the proposed SCSSA-CNN-BiLSTM model. This robustness mainly stems from the model’s ability to learn and generalize the underlying thermal-mechanical relationships, rather than merely memorizing the data patterns of specific operating conditions. When dealing with speed variations, the performance of this model indicates that it effectively separates the influence of thermodynamics from the motion state of the shaft. Although the speed determines the magnitude and rate of heat generation, the fundamental physical principles governing heat transfer and subsequent thermal deformation remain consistent. Through its spatial-temporal feature extraction, the model has learned these invariant physical laws, thereby being able to accurately infer untested speeds within the operational range from the training speed.

Regarding the performance on different spindles, the maximum model prediction error observed was between ± 4.6 μm and ± 5.0 μm. This confirms that the model performs well across different spindles. Although there may be inevitable minor differences in cooling conditions, assembly tolerances, or surrounding structures, the spindles of the same model and construction are generally consistent in this regard. The model successfully ignores these unique noise factors and focuses on the main, shared thermal characteristics. This ability to standardize subtle differences between spindles is crucial for practical applications, as it indicates that a well-trained single model has the potential to be deployed in multiple identical machines without the need for extensive individual recalibration, thereby significantly reducing the engineering cost of thermal error compensation for the entire fleet.

## Discussion

### Implementation path and expansion plan for the model

To achieve the stable deployment of the thermal error compensation model in the actual industrial environment, the first step is to install a temperature sensor array on the target CNC machine to collect sufficient and representative thermal characteristic data. Subsequently, the proposed model is used for offline training to obtain optimized parameters. The trained model will be embedded as a thermal error prediction module into the CNC system. This module can be extended through API interfaces or user-defined NC codes to achieve real-time communication with the CNC control system. After inputting the real-time collected temperature data into the model, the system can obtain the thermal error compensation value and provide it in real time to the control unit, thereby achieving dynamic compensation of the tool path and the origin of the coordinate system. At the system integration level, this model can rely on programmable logic controllers (PLC) or dedicated compensation hardware for real-time calculation and transmit the compensation instructions to the CNC system through digital I/O, EtherCAT, and other fieldbus protocols to ensure the reliability and stability of the compensation process.

At the same time, to further expand to different machines, we plan to adopt the transfer learning strategy to achieve efficient model expansion: using the trained model from the source machine (1200MSY) as the pre-training base network, when deploying to a new model machine, only a small amount of thermal data of the new machine needs to be collected to fine-tune the last layer or some layers of the model, which can quickly adapt without having to retrain from scratch. This method will significantly reduce the engineering cost of data collection and model calibration on multiple machines and provide an efficient and feasible solution for large-scale engineering applications.

### Limitations and future work

Although the proposed SCSSA-CNN-BiLSTM model demonstrates excellent accuracy and strong generalization ability on different speeds and spindles within the same machine, this research constitutes a basic stage of the study. It is necessary to acknowledge several important limitations inherent in this stage. These limitations not only define the current working boundaries of our work but also clearly indicate the direction for our future research work.

(1)This research project aims to further integrate multiple sensing signals such as temperature, vibration, spindle current, and cutting force, and construct a thermal error collaborative modeling framework that integrates multiple physical information. By extracting complementary features of the thermodynamic behavior during dynamic machining, the model’s prediction accuracy and robustness under varying conditions will be enhanced. Additionally, future work will focus on lightweight model design, neural architecture search, and hardware-in-the-loop testing, with the goal of optimizing the computational efficiency and inference speed of the system, providing a technical foundation for real-time embedded deployment.

(2)This model was developed and validated only on a specific machine model (1200MSY). Its performance in comparison with other machine architectures and configurations has not yet been empirically verified. Moreover, the selection of temperature-sensitive points relies on data-driven methods (K-means clustering and grey relational analysis), and there is a lack of confirmation of the true representativeness of the sensitive points through physical models (such as finite element analysis - FEA) or thermal imaging. We also plan to use techniques such as SHAP or LIME for analysis, to quantify feature importance, and to make the decision-making process of the model transparent and trustworthy for engineers.

(3)All the experiments were conducted in a controlled laboratory environment. The performance of this model has not yet been evaluated under various real production conditions, such as different cutting loads, intermittent operation, the presence of coolant and dust, or significant macroscopic environmental changes (for example, large diurnal or seasonal fluctuations in workshop temperatures). Therefore, its long-term stability against sensor drift, gradual mechanical wear, and long-term (several months) performance degradation has not yet been assessed.

## Conclusion

In order to solve the problems in the thermal error modeling of CNC machine tools, such as insufficient mining of timing features, limited model generalization ability, and verification at the fixed speed of a single spindle, this paper uses 1200MSY dual-spindle turning and milling machine tools As the research object, SCSSA-CNN based on K-means clustering (K-means) combined with grey correlation analysis based on temperature measurement points was established -BiLSTM Thermal Error Prediction Model for Dual-Spindle Turning and Milling Machines, and the following conclusions are obtained:

1. Comparison between SCSSA-CNN-BiLSTM dual-spindle thermal error prediction model and commonly used thermal error algorithms, SCSSA- The root mean square error (RMSE) predicted by the CNN-BiLSTM dual-spindle model was reduced by up to 64.92%;The absolute percentage error (MAE) was reduced by up to 66.50%. Indicates the prediction accuracy of the model used.
2. The proposed SCSSA-CNN-BiLSTM dual-spindle thermal error prediction model can achieve good prediction results under multi-speed conditions. The thermal error prediction of the double spindle also has high accuracy, which provides a better high-precision and strong generalization solution for the collaborative thermal error compensation of the double-spindle turning and milling machine.

## Supplementary Information

Below is the link to the electronic supplementary material.

Supplementary Material 1

## Data Availability

All the data generated or analyzed during this research are included in this article [and its supplementary information file].
